# Marathon running transiently depletes the myocardial lipid pool

**DOI:** 10.14814/phy2.14543

**Published:** 2020-09-01

**Authors:** Vincent L. Aengevaeren, Martijn Froeling, Sandra van den Berg‐Faay, Melissa T. Hooijmans, Jithsa R. Monte, Gustav J. Strijkers, Aart J. Nederveen, Thijs M.H. Eijsvogels, Adrianus J. Bakermans

**Affiliations:** ^1^ Radboud Institute for Health Science, Department of Physiology Radboud University Medical Center Nijmegen The Netherlands; ^2^ Radboud Institute for Health Sciences, Department of Cardiology Radboud University Medical Center Nijmegen The Netherlands; ^3^ Department of Radiology University Medical Center Utrecht Utrecht The Netherlands; ^4^ Department of Radiology and Nuclear Medicine Amsterdam University Medical Centers, University of Amsterdam Amsterdam The Netherlands; ^5^ Biomedical Engineering and Physics Amsterdam University Medical Centers, University of Amsterdam Amsterdam The Netherlands

**Keywords:** endurance exercise, lipid metabolism, myocardium, proton magnetic resonance spectroscopy, triglycerides

## Abstract

Lipids, stored as intracellular triacylglycerol droplets within the myocardium, serve as an important source of energy, particularly in times of prolonged increased energy expenditure. In only a few studies, the acute effects of exercise on such ectopic myocardial lipid storage were investigated. We studied the dynamic behavior of the myocardial lipid pool in response to completing the 2017 Amsterdam Marathon using proton magnetic resonance (MR) spectroscopy (^1^H‐MRS). We hypothesized that the prolonged increased myocardial energy demand of running a marathon could shift the balance of myocardial triacylglycerol turnover from triacylglycerol synthesis toward lipolysis and mitochondrial fatty acid β‐oxidation, and decrease the myocardial lipid pool.

We employed two 3 Tesla MR systems in parallel to noninvasively examine endurance‐trained healthy men (*n* = 8; age 50.7 [50.1–52.7] y) at 1 week prior (baseline), <6 hr after finishing the marathon (post‐marathon), and 2 weeks thereafter (recovery). Exercise intensity was 89 ± 6% of the age‐predicted maximal heart rate, with a finish time of 3:56 [3:37–4:42] h:min. Myocardial lipid content was 0.66 [0.58–0.87]% of the total myocardial water signal at baseline, was lower post‐marathon (0.47 [0.41–0.63]% of the total myocardial water signal), and had restored to 0.55 [0.49–0.83]% of the total myocardial water signal at recovery, representing a transient marathon running‐induced depletion of 29 ± 24% (*p* = .04). The magnitude of this myocardial lipid pool depletion did not correlate with exercise intensity (*r *= −0.39; *p* = .39), nor with marathon finishing time (*ρ* = 0.57; *p* = .15).

Our data show that prolonged high‐intensity exercise can induce a transient depletion of the myocardial lipid pool, reinforcing the dynamic nature of ectopic triacylglycerol storage under real‐life conditions of extreme endurance exercise.

## Graphical abstract

The acute effects of exercise on ectopic myocardial lipid storage have not been investigated in a real‐life endurance exercise scenario. We studied the dynamic behavior of the myocardial lipid pool in response to marathon running with proton MR spectroscopy. Using two MR systems in parallel, we examined eight endurance‐trained men within 6 hr of completing a marathon, and compared results with baseline and recovery measurements. Our data demonstrate a transient high‐intensity exercise‐induced depletion of the myocardial lipid pool.

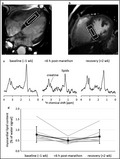

## 1 INTRODUCTION

Lipids serve as an important source of energy, particularly in times of metabolic stress or prolonged increased energy expenditure such as during endurance exercise (van Loon, 2004). During prolonged fasting or exercise, lipolysis of triacylglycerols in adipose tissue releases free fatty acids for oxidative metabolism in muscles, including the myocardium. Caloric restriction (van der Meer et al., 2007), overnight fasting (Ith, Stettler, Xu, Boesch, & Kreis, 2014), or prolonged 48‐hr fasting (Reingold et al., 2005) temporarily elevates the levels of circulating free fatty acids, and leads to *increased* ectopic triacylglycerol storage in the form of intracellular lipid droplets within the myocardium. In only a few studies, the acute effects of exercise on myocardial lipid storage were investigated (Loher, Kreis, Boesch, & Christ, 2016). A single 2‐hr bout of cycling exercise under fasted conditions, but not while glucose‐fed, was shown to induce a threefold increase in plasma free fatty acid concentrations with a concomitant increase in myocardial lipid content in 11 untrained men (Bilet et al., 2011). Another study in 10 healthy men demonstrated a *decrease* of ectopic myocardial lipid content after a 2‐hr bout of moderate‐intensity aerobic bicycling exercise, but those participants were preloaded with a standardized fat‐rich diet for 3 days prior to exercise examination (Bucher et al., 2014). Those studies emphasize the dynamic nature of myocardial lipid metabolism (Taegtmeyer & Harmancey, 2008), but were conducted in a controlled laboratory setting with distinct nutritional interventions (fasting or glucose ingestion (Bilet et al., 2011), and fat loading (Bucher et al., 2014)) and involved exercise for only 2 hr at moderate intensity without long‐term follow‐up measurements after recovery. As such, the dynamic behavior of intracardiomyocellular lipid pools following prolonged high‐intensity exercise under representative conditions of real‐life activity (i.e., with ad libitum nutrition and training regime) remains unknown (Loher et al., 2016).

We performed a pragmatic study to investigate the dynamic behavior of the intracardiomyocellular lipid pool in response to a marathon run. We hypothesized that the prolonged increased myocardial energy demand of running a marathon could shift the balance of myocardial triacylglycerol turnover from triacylglycerol synthesis toward lipolysis and mitochondrial fatty acid β‐oxidation (van Loon, 2004), and decrease the myocardial lipid pool. To test this, we noninvasively measured the myocardial lipid content with proton magnetic resonance (MR) spectroscopy (^1^H‐MRS) in healthy endurance‐trained men before, directly after, and 2 weeks after finishing the 2017 Amsterdam Marathon. In addition, we explored any potential associations between (exercise‐induced changes in) myocardial lipid pools and exercise characteristics.

## 2 MATERIALS AND METHODS

This observational study (Aengevaeren et al., 2020) was approved by the local Ethics Committee (NL61873.018.17; Academic Medical Center, University of Amsterdam, Amsterdam, The Netherlands). All subjects provided written informed consent. We recruited 12 male participants aged ≥45 years of the 2017 Amsterdam Marathon. Eligible subjects must have had completed at least one marathon previously. Exclusion criteria were known cardiovascular diseases or risk factors. Subjects underwent cardiac MR examinations at 1 week prior to the marathon (baseline), within 6 hr after finishing the marathon (post‐marathon), and 2 weeks thereafter (recovery). Venous blood samples were collected within 1 hr pre‐marathon and at the time of MR examination post‐marathon, and were processed immediately according to standard blood panel assays.

### 2.1 ^1^H‐MRS protocol

**FIGURE 1:**
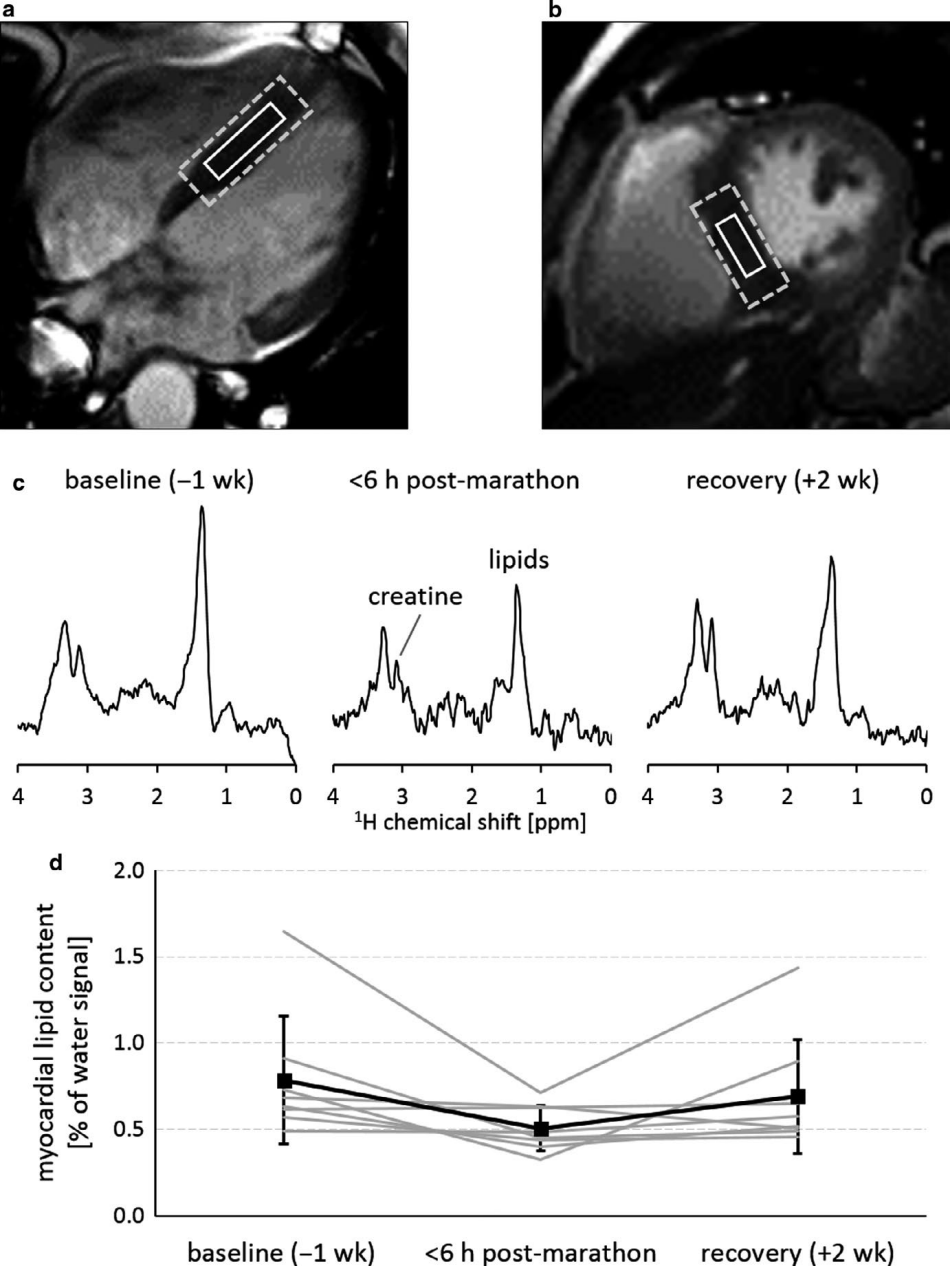
Localized proton magnetic resonance spectroscopy (^1^H‐MRS) of myocardial lipid content in healthy male marathon runners at 3 Tesla. A voxel (solid white box) was carefully positioned in the septum (a,b) and was enclosed by the shim volume (dashed box). Spectra were acquired 1 week before the marathon at baseline, directly post‐marathon, and after 2 weeks of recovery, showing distinct signals of myocardial creatine and lipids (c). Myocardial lipid content, quantified relative to the total water signal, was depleted after completing the marathon, and restored after 2 weeks of recovery (d). Individual data for each participant are shown in grey, with group mean values (*n* = 8) indicated by the black squares. Error bars indicate standard deviation.

The MR examinations were conducted on two 3 Tesla MR systems (Ingenia; Philips, Best, The Netherlands). Each subject was scanned on the same system for all time points to mitigate any system‐specific variations between measurements. After standard cine imaging of left‐ventricular long‐axis (Figure 1a) and short‐axis views (Figure 1b), localized ^1^H‐MR spectra (Figure 1c) were acquired from a 10 × 20 × 35 mm^3^ voxel positioned in the septum (Figure 1a,b) using a single‐voxel point‐resolved spectroscopy (PRESS) sequence as described previously (de Heer, Bizino, Lamb, & Webb, 2016). Acquisitions were ECG‐triggered at 200 ms after R‐wave detection, and gated to end‐expiration. Water‐suppressed spectra (multiple optimizations insensitive suppression train, MOIST; bandwidth, 220 Hz) were obtained at an echo time of 40 ms and a repetition time of >6 s to avoid partial saturation effects for signal quantification. Other parameters were as follows: triacylglycerol‐methylene at 1.30 ppm on‐resonance; number of acquisitions, 64; number of points, 1,024; bandwidth, 1,500 Hz. Acquisitions of water‐suppressed spectra were interleaved with eight acquisitions of unsuppressed spectra from the same voxel (water at 4.7 ppm on‐resonance) to obtain the fully relaxed water signal (TR, >9 s) as a quantification reference.

Spectral fitting was performed in the time‐domain using AMARES in jMRUI (Vanhamme, van den Boogaart, & Van Huffel, 1997). The (phospho)creatine‐methyl resonance was used as internal chemical shift reference at 3.02 ppm. Lipid signals from triacylglycerols (Griffin, Williams, Sang, & Nicholson, 2001) between 0.85 ppm and 2.7 ppm and the triacylglycerol olefinic protons at 5.3 ppm were fitted to Gaussian line shapes. The unsuppressed water signal was fitted to a Lorentzian line shape and used as a quantification reference. Myocardial lipid content was quantified as the percentage of the sum of the lipid signal amplitudes over the unsuppressed water signal amplitude measured in the same voxel.

### 2.2 Statistical analyses

Statistical analyses were performed using SPSS 24.0 (SPSS, Inc.). Variables were visually inspected for normality, and checked for kurtosis and skewness. Continuous variables are reported as mean ± standard deviation (*SD*) when normally distributed, or as median [interquartile range] when not normally distributed. Repeated‐measures analysis of variance was used to compare normally distributed continuous variables between baseline, post‐marathon, and at recovery. Related‐samples Friedman's two‐way analysis of variance by ranks was used when continuous variables were not normally distributed. Relations between variables were tested with Pearson's *r* or Spearman's *ρ* (if one or both variables were not normally distributed) correlation coefficients. Differences between pre‐ and post‐marathon blood panel results were tested with the Wilcoxon signed‐rank test. The level of significance was set at *p* < .05.

## 3 RESULTS

One participant did not complete the marathon due to acute knee injury and was excluded from the study. Of the remaining 11 marathon finishers, one subject had a metal plate implant on the left clavicle that interfered particularly with localized ^1^H‐MRS on all time points. Another subject could not lie sufficiently still during the post‐marathon MR exam, and for a third participant the ECG signal was of poor quality, hampering the successful acquisition of localized ^1^H‐MR spectra in these subjects. Thus, a complete dataset for all time points was available for eight subjects (age, 50.7 [50.1–52.7] y; height, 1.80 [1.72–1.82] m; body mass, 75.2 ± 5.9 kg; body mass index, 23.7 ± 1.3 kg/m^2^; systolic blood pressure, 126 ± 9 mmHg; diastolic blood pressure, 75 ± 6 mmHg). These participants had performed lifelong exercise training for 18.0 [15.0–25.3] METh/week and had completed 11 [2–15] marathons in the past. Their median finish time of the 2017 Amsterdam Marathon was 3:56 [3:37–4:42] h:min. Mean heart rate during the race was 153 ± 12 beats/min, which equates to an exercise intensity at 89 ± 6% of the age‐predicted maximal heart rate (Tanaka, Monahan, & Seals, 2001).

Body mass was lower post‐marathon (−2.7 ± 0.5%; *p* = .001) and had returned to baseline values at recovery. After finishing the marathon, total blood cholesterol had decreased by 5.0 ± 3.5% (*p* = .02; Table 1). Post‐marathon blood glucose concentrations were higher than before the start of the marathon (+43.4 ± 33.8%; *p* = .02), which were paralleled by elevated insulin levels (*p* = .04), and are likely explained by ad libitum nutrition during and after the race. Plasma free fatty acids (*p* = .34) and triacylglycerol concentrations (*p* = .07) pre‐ and post‐marathon were similar.

**TABLE 1:** Blood panel results of the marathon finishers obtained just prior to the start of the marathon and at the time of post‐marathon MR examination

Male marathon runners (*n* = 8)	1 hr pre‐marathon	<6 hr post‐marathon	*p*‐value
glucose [mM]	5.1 [4.6–6.1]	7.8 [7.1–8.5]	**.02**
insulin [pM]	27 [16–72]^a^	250 [74–398]	**.04**
HbA1c [mmol/mol]	33.5 [32.3–36.7]^a^	—	—
total cholesterol [mM]	6.1 [4.7–6.3]	5.8 [4.4–5.9]	**.02**
HDL cholesterol [mM]	1.4 [1.3–2.0]	1.5 [1.3–2.0]	.09
LDL cholesterol [mM]	3.5 [2.7–4.0]	3.4 [2.6–3.9]	.16
triacylglycerols [mM]	1.1 [0.9–2.0]	0.9 [0.6–1.3]	.07
free fatty acids [mM]	0.12 [0.12–0.13]	0.12 [0.09–0.46]	.34

Note

Data are presented as median [interquartile range], with *p*‐values reported for Wilcoxon signed‐rank tests. The level of significance was set at p < .05, represented in bold values.

Hb, hemoglobin; HDL, high‐density lipoprotein; LDL, low‐density lipoprotein.

^a^ Measured at baseline.

**FIGURE 2:**
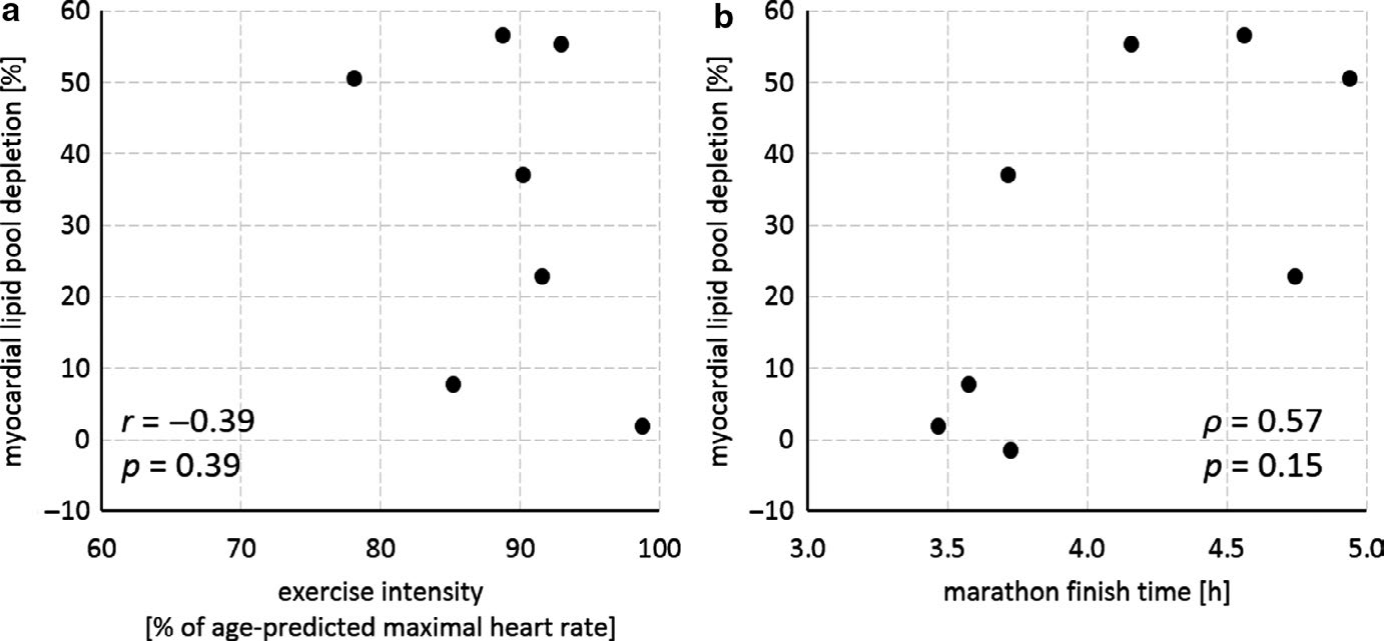
The exercise‐induced depletion of the myocardial lipid pool did not correlate with exercise intensity (Pearson's *r*; a), nor with marathon finishing time (Spearman's *ρ*; b). Myocardial lipid pool depletion was defined as the decrease in myocardial lipid content measured within 6 hr post‐marathon relative to baseline values, quantified in healthy male marathon runners (*n* = 8) using proton magnetic resonance spectroscopy (^1^H‐MRS) at 3 Tesla. Heart rate monitoring throughout the course of the marathon run was incomplete for one participant, and an accurate estimate of exercise intensity (*n* = 7; a) is missing for that participant.

Myocardial lipid content (Figure 1d) was 0.66 [0.58–0.87]% of the total myocardial water signal at baseline, and had decreased by 0.18 [0.03–0.43]% of the total myocardial water signal at post‐marathon (0.47 [0.41–0.63]% of the total myocardial water signal), representing a marathon running‐induced depletion of the myocardial lipid pool by 29 ± 24% (*p* = .04). The magnitude of depletion correlated positively with baseline myocardial lipid content (*ρ* = 0.81; *p* = .02), indicating that depletion was more pronounced in participants with a larger myocardial lipid pool. Values had restored to 0.55 [0.49–0.83]% at recovery. The magnitude of myocardial lipid pool depletion did not correlate with exercise intensity (*r* = −0.39; *p* = .39; Figure 2a), nor with marathon finishing time as a measure of exercise duration (*ρ* = 0.57; *p* = .15; Figure 2b).

## 4 DISCUSSION

In this study, we noninvasively measured the myocardial lipid content in eight experienced male marathon runners within 6 hr after finishing a marathon using ^1^H‐MRS of the heart. We compared these post‐marathon results with measurements performed at baseline and after 2 weeks of recovery. Our results indicate that prolonged high‐intensity exercise can induce a transient depletion of the myocardial lipid pool, demonstrating the dynamic nature of ectopic triacylglycerol storage under real‐life conditions of extreme endurance exercise.

Our data show that marathon running depletes the myocardial lipid pool in endurance‐trained men. The observed depletion of nearly 30% is higher than the 17% reduction that was found in healthy men who were preloaded with a fat‐rich diet for 3 days prior to a 2‐hr bout of moderate‐intensity (50%–60% of the maximal oxygen uptake rate) aerobic bicycling exercise (Bucher et al., 2014). Our observation suggests that the degree of exercise‐induced myocardial lipid pool depletion may be exercise dose‐dependent, with the high exercise intensity and prolonged duration of marathon running leading to greater decreases of the myocardial lipid pool. However, any significant correlations between lipid pool depletion and exercise intensity or duration could not be established in the present study, possibly because of the relatively narrow distribution of exercise intensity (range 78%–99% of the age‐predicted maximal heart rate) within our small cohort of experienced and endurance‐trained men. In apparent contrast to our results and previous work (Bucher et al., 2014), one study (Bilet et al., 2011) reported an increase of nearly 70% in myocardial lipid content just 4 hr after performing a 2‐hr bout of moderate‐intensity exercise in the fasted state. This effect was absent when participants ingested glucose prior to and during exercise (Bilet et al., 2011). Differences in the nutritional state, diurnal variations (Ith et al., 2014), the intensity of the exercise intervention, and the time span between cessation of exercise and MR examination likely explain any differential findings of the present study and the very few other studies available to date.

The recovery of myocardial lipid content to baseline values indicates that exercise‐induced depletions are transient, and reinforces the dynamic nature of myocardial lipid metabolism, particularly in endurance‐trained athletes. It is known that ectopic lipid storage in skeletal muscle is elevated both in type 2 diabetes patients as well as in well‐trained endurance athletes, but unlike patients with type 2 diabetes, athletes are highly insulin sensitive and their intracardiomyocellular lipid pool functions as a dynamic fuel store during exercise (van Loon, 2004). In absence of a control group, our baseline measurements cannot establish whether the myocardial ectopic lipid storage is analogously elevated in this small cohort of marathon runners. Reported values for myocardial lipid content (0.6 ± 0.4%) measured with essentially the same ^1^H‐MRS protocol as we used here indicate that the present baseline values for endurance‐trained men lie well within the normal range (de Heer et al., 2016). Notably, the magnitude of marathon running‐induced depletion correlated with baseline myocardial lipid content, suggesting that an elevated ectopic lipid storage in the heart of these athletes is readily recruited for mitochondrial fatty acid β‐oxidation during prolonged times of increased myocardial energy demand (Jacobs & Lundby, 2013; van Loon, 2004).

Similar to the few reports on the acute effects of exercise on ectopic lipid storage (Bilet et al., 2011; Bucher et al., 2014), we used ^1^H‐MRS to measure the myocardial lipid content. It is a nonionizing imaging modality, and uniquely well‐suited for noninvasive longitudinal assessments of the lipid pool in human tissues (Loher et al., 2016). However, it is also a complex technique that requires specific resources and expertise, and is therefore typically not included in a standard clinical MR exam, particularly not in cardiac examinations. Moreover, MR exams are relatively time‐consuming, and MR capacity can be a limiting factor when aiming to investigate multiple participants directly after a high‐intensity exercise effort, particularly in this real‐life scenario of marathon running. Despite these tremendous practical challenges, and by using two MR systems in parallel, we managed to obtain complete datasets for eight marathon runners at baseline, directly post‐marathon, and after recovery. Nonetheless, the cohort size of this study was inherently small, and more work is needed to expand the sample size in order to establish any relations between dynamic changes in myocardial lipid content and exercise intensity or duration.

In conclusion, we demonstrate that, in a real‐life endurance exercise scenario, the intracardiomyocellular lipid pool is highly dynamic, and suggest that prolonged high‐intensity exercise can constitute an energy‐demanding condition that is sufficient to induce a rate of myocardial lipolysis and mitochondrial fatty acid β‐oxidation that exceeds lipid storage through triacylglycerol synthesis. Further studies are warranted to establish whether an exercise‐induced reduction of the myocardial lipid pool, and the magnitude of this response, can have an effect on heart function.

## DISCLOSURES

The authors declare no potential conflict of interest.

## AUTHOR CONTRIBUTIONS

VLA, AJN, TMHE, and AJB conceived and designed research; VLA, MF, SvdBF, MTH, JRM, TMHE, and AJB performed experiments; VLA and AJB analyzed data; VLA, TMHE, and AJB interpreted results of experiments; AJB prepared figures; VLA, TMHE, and AJB drafted manuscript; VLA, MF, SvdBF, MTH, JRM, GJS, AJN, TMHE, and AJB edited and revised manuscript; VLA, MF, SvdBF, MTH, JRM, GJS, AJN, TMHE, and AJB approved the final version of manuscript.
